# An innovative method of the vertical coupling effect improvement to the tandem Cu(In, Ga)Se_2_/perovskite solar cells using Ag cluster nanostructures

**DOI:** 10.1038/s41598-024-64822-x

**Published:** 2024-06-15

**Authors:** Parisa Zarerasouli, Fatemeh Aghaei, Hamid Bahador

**Affiliations:** 1 Department of Electrical and Computer Engineering, University of Mohaghegh Ardabili, Ardabil, Iran; 2 Faculty of Electrical and Computer Engineering, University of Tabriz, Tabriz, Iran

**Keywords:** Materials for optics, Theory and computation, Materials science

## Abstract

The efficiency of double-junction CIGS/Perovskite-based solar cells has significantly improved through recent research. This study presents a new plasmonic structure for these optical devices, utilizing cluster nanostructures to increase photon absorption between 650 and 1137 nm wavelength ranges. The proposed nanostructure includes two vertically coupled silver nanoparticles embedded at the center of the bottom active layer (CIGS) that absorb most of the incoming light to CIGS within the active layer. The electric field produced by the coupling of the nanoparticles has a superior performance. To analyze the effect of nanoparticle coupling on CIGS/Perovskite solar cell performance, evaluated the short-circuit current density and power conversion efficiency for single and cluster nanostructures with a single nanoparticle in the middle of CIGS. The structures with a single nanoparticle displayed J_sc_ = 16.89 mA cm^−2^ and PCE = 31.76%, while the cluster nanostructure represents J_sc_ = 19 mA cm^−2^ and PCE = 35.81%. Not only did the use of the cluster nanostructure significantly improve absorption and performance compared to the bare case, but it also exhibited a suitable improvement compared to the single nanoparticle.

## Introduction

In recent years, solar cells have become increasingly popular as a cleaner alternative to fossil fuels to limit environmental harm^1–3^. However, they have limitations such as high manufacturing costs and low efficiency^4^. One solution to these issues is developing thin-film and efficient solar cells. Tandem solar cells, which consist of multiple active layers with different band gaps, offer a promising solution^5^. This not only improves photon absorption but also enhances the absorption spectrum of the solar cell^6,7^. To further improve efficiency and cost-effectiveness, double-junction tandem structures are commonly used.

Lately, there has been a lot of interest in double-junction solar cells that use CIGS/Perovskite due to their unique advantages^5^. These solar cells consist of a CIGS-based bottom active layer and a perovskite-based top active layer. Both layers have high absorption coefficients, direct and tunable band gaps (1–1.7 eV for CIGS, 1.17–3.1 eV for perovskite), and long carrier emission lengths. Furthermore, they require low thicknesses to absorb light, which makes them more cost-effective to manufacture^8,9^. Although their efficiency was previously not very high (about 15.9% in 2015)^10^, recent research has significantly improved their efficiency to 19.29% by utilizing nano prisms to increase light reflection and trapping and substituting new layers^11^. In 2019, one study achieved an impressive efficiency of 31.13% by optimizing layer thickness and using anti-reflection coating^12^.

Despite significant advancements in this field, it is important to acknowledge that there is still room for improvement in the efficiency of CIGS/Perovskite-based solar cells. One promising method that has not received enough attention is the plasmonic light trapping technique, which involves using noble metal nanoparticles like silver (Ag), gold (Au), copper (Cu), and aluminum (Al) to improve performance^13–15^. However, it is worth noting that silver has shown to be the most effective out of these metals, especially when embedded in the active layer, due to lower absorption losses and ultimately better efficiency^16,17^.

It's worth noting that nanoparticles can create an electromagnetic field by concentrating light at a specific frequency, known as the resonance frequency. This occurs because of the collective oscillation of free electrons of metals, called surface plasmon resonance (SPR)^18,19^. Additionally, adding two or more nanoparticles to the active layer at a suitable distance apart can greatly increase efficiency^20^. This is due to the formation of nanoparticles' coupling and the overlapping of electric fields, resulting in amplified field intensity and light scattering in various directions. As a result, this is expected to increase the net absorption coefficient and the likelihood of photon absorption, surpassing other scenarios^13^.

As we delve deeper into the world of solar cells, it becomes apparent that the positioning of nanoparticles in specific layers can significantly impact their efficiency. For example, incorporating nanoparticles into Perovskite layers can boost the absorption of high-energy photons, while using them in CIGS layers can enhance the absorption of low-energy photons. However, utilizing nanoparticles in both active layers can increase the cost of solar cells. This is why researchers are working towards developing an affordable and effective CIGS/Perovskite solar cell that prioritizes enhancing weak light absorption at longer wavelengths. One of the methods being studied involves integrating nanoparticles in clusters within the bottom active layer (CIGS).

## Proposed structure

**Figure 1:**
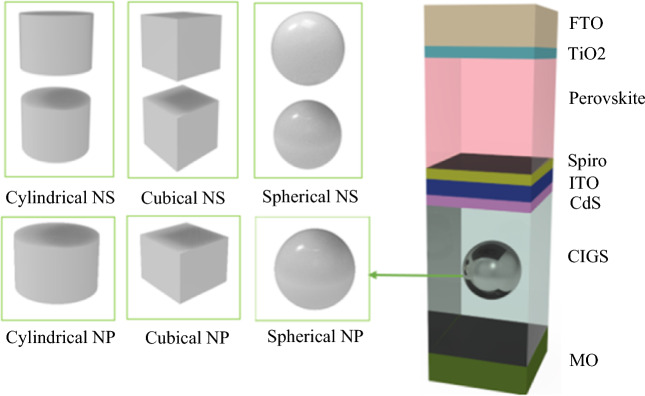
3D schematic representation of the proposed structure for tandem CIGS/Perovskite solar cell for different geometric NPs.

Displayed in Fig. 1 is a highly efficient and fundamental CIGS/Perovskite structure, composed of eight layers arranged in a specific order. These layers, namely Mo, CIGS, CdS, ITO, Spiro, Perovskite, TiO2, and FTO, each serve a specific function within the structure. While Mo functions as the back contact, FTO operates as the anti-reflector coating. Table 1 provides information on the thickness of each layer. It should be noted that the unit cell in this structure has a 300 nm period.

**Table 1:** Simulation properties of layers.

Parameter	Description	Material	Value (nm)
t_bc_	Back contact	Mo	60
t_bot, act_	Bottom active layer	CIGS	300
t_buf_	Buffer layer	CdS	10
t_int_	Intermediate layer	ITO	30
t_htl_	Hole transfer layer	Spiro	10
t_top, act_	Top active layer	Perovskite	200
t_etl_	Electron transfer layer	TiO_2_	10
t_ar_	Optical transparent cathode	FTO	70
a	Period of structure	–	300
*l*	Dimension of NPs	Ag	20 < *l* < 100

When designing double-junction tandems, it's essential to consider the band gap range of each layer for optimal performance. The top active layer should have a band gap between 1.5 and 1.8 eV, while the bottom layer should range from 0.9 to 1.2 eV^12^. In this work, methylammonium lead iodide (CH_3_NH_3_PbI_3_) is used as the top absorber because of its 1.5 eV band gap. Theoretical research suggests that a bottom active layer with a band gap close to 1 eV produces better results^5^. Therefore, we opted for CIGS with a band gap of 1.09 eV as the closest available value^21^.

The layer thicknesses are optimized, ranging from 5 to $\lambda$/4 nm, as shown in Table 1. It's essential to note that increasing the thickness of active layers significantly increases the current difference between top and bottom layers, while the plasmonic effect of metal nanoparticles decreases with high thickness.

In this research, the methods to enhance the performance of solar cells have been explored and led to examining single nanoparticles within a plasmonic structure. A thorough study was conducted on nanoparticles of various shapes, such as cubical, spherical, and cylindrical, to establish the ideal size (*l*) for placement at the center of CIGS. The objective was to factor in absorption losses and determine the size that would yield maximum efficiency. Currently, our focus is on studying coupling vertically phenomenon, rather than horizontally, to minimize losses. To achieve this, only two nanoparticles are being utilized with different geometries, positioned vertically and spaced 5 nm apart.

## Modeling and simulation method

The absorption spectrum was analyzed using a plane wave light source with a working wavelength range of 300 to 1137 nm, positioned on top of the structure through the FDTD method. The solar spectrum's minimum wavelength and the minimum band gap of the active layers, which was 1.09 eV for CIGS, determined the operation wavelength^22^. The vertical direction had perfectly matched layer (PML) boundary conditions, while the direction of periodicity of the unit cell had periodic boundary conditions to guarantee accuracy. The absorption spectrum was displayed using the Eq. (1) software solution^23^.

1 $$P_{abs} = - \frac{1}{2}\omega \varepsilon_{0} \left| {E(r,\omega )} \right|^{2} {\text{Im}} (\varepsilon (r,\omega ))$$

The absorption spectrum is a crucial factor in evaluating solar cell performance. This spectrum is governed by a complex equation that considers various critical factors, such as angular frequency ($\omega$), vacuum permeability ($\varepsilon_{0}$), electric field (E), dielectric constant ($\varepsilon$), and position (r). Increasing the intensity of the electromagnetic field is a surefire way to improve absorption, leading to an undeniable rise in the short-circuit current density. In the range of 300–1137 nm, the short-circuit current density relationship is as follows^24^:

2 $$J_{sc} (\lambda ) = q\int_{300}^{1137} {\frac{\lambda }{hc}} \frac{{P_{abs} (\lambda )}}{{P_{in} (\lambda )}}I_{AM1.5} (\lambda )d\lambda$$

Upon inputting the given values into the equation, it is evident that q equals 1.602 × 10^−19^C, h equals 4.1356 × 10^−15^ eV s^−1^, and c equals 3 × 10^8^ ms^−1^. It is crucial to note that I_AM1.5_ represents the photon flux density of the AM1.5 solar spectrum, while P_in_ equates to 1000 mW m^−2^ according to the AM1.5 solar spectrum. This equation unequivocally suggests that an increase in photon absorption within the active layer will undoubtedly result in a significant rise in the short-circuit current density of the solar cell. Furthermore, it is imperative to estimate the minimum current as the short-circuit current density of tandem structures as per Eq. (3)^25^.

3 $$J_{sc,Tandem} = \min (J_{sc,top} ,J_{sc,bottom} )$$

When exploring the generation of photons and the interaction between particles, it's important to take into account the rate of photon generation (Eq. 4) and the electric field^22^.

4 $$G = - \frac{\pi }{h}\left| {E(r,\omega )} \right|^{2} \left\{ {\varepsilon (r,\omega \left. ) \right\}} \right.$$

The strength of the electric field plays a vital role in evaluating the effectiveness of solar cells. The performance of the solar cell can be improved by increasing the electric field intensity through metal nanoparticle coupling in cluster nanostructures. Additionally, the open circuit voltage (V_oc_)^26^ is a crucial parameter for analyzing recombination (Eq. 5). This is because the open circuit voltage is tied to the dark saturation current density (J_0_). Consequently, an improvement in the open circuit voltage would suggest a reduction in the solar cell's recombination losses^18^.

5 $$V_{oc} = \frac{nkT}{q}\ln \left( {\frac{{J_{sc} }}{{J_{0} }} + 1} \right)$$

The thermal voltage (*kT/q*) in this equation must always be 25.9 mv. CIGS has a fixed value of J_0_ at 3.4 × 10^–13^, whereas for perovskite, it is 1.23 × 10^–19^^27^. When determining the open circuit voltage for tandem structures, it is crucial to utilize Eq. (6) which involves adding the voltages of the top and bottom solar cells^25^.

6 $$V_{oc,Tandem} = V_{oc,top} + V_{oc,bottom}$$

To accurately calculate the fill factor using Eq. (7), the open circuit voltage must be determined^28^.

7 $$FF = \frac{{\frac{{V_{oc} q}}{nkT} + \ln \left( {\frac{{V_{oc} q}}{nkT} + 0.72} \right)}}{{\frac{{V_{oc} q}}{nkT} + 1}}$$

Lastly, the power conversion efficiency (PCE) must be calculated to identify the most optimal structure as following^29^:

8 $$PCE(\% ) = \frac{{J_{{sc,{\text{Tan}} dem}} V_{{oc,{\text{Tan}} dem}} FF}}{{P_{in} }}$$

Assuming shunt resistance (r_sh_) equal to (V_oc_/J_sc_), and voltage (V) sweep from 0 to V_oc_, the current–voltage (J–V) characteristics can be calculated by Eq. (9)^30,31^:

9 $$J = J_{sc} - J_{0} (e^{(qV/(nkT))} - 1) - (V/r_{sh} )$$

## Simulation results and discussion

**Figure 2:**
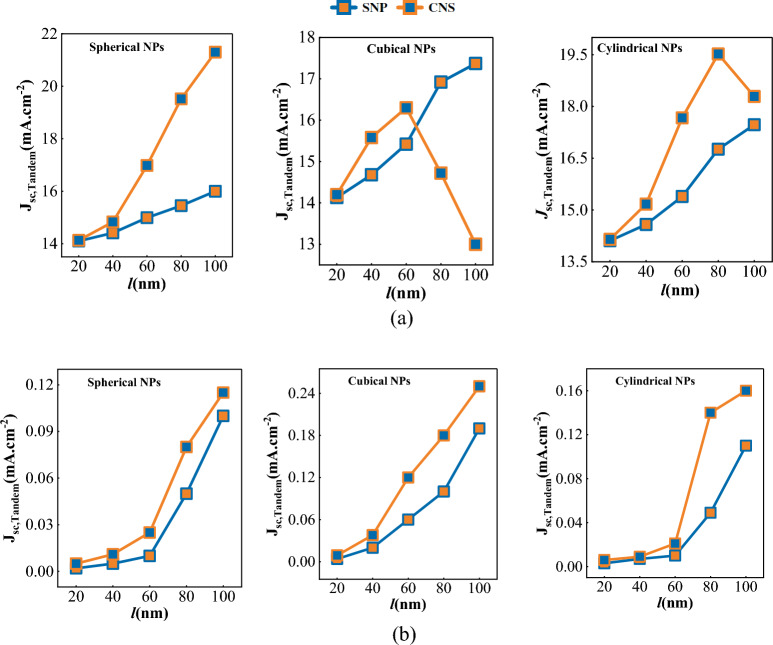
(**a**) Short circuit current density of CIGS/perovskite tandem structure with single nanoparticle and cluster nanostructure, and (**b**) the maximum absorption losses of single nanoparticles and cluster nanostructures for different geometries and sizes (*l*) of metal particles. (Here, *l* states the dimensions of cubical, cylindrical and diameters of spherical NPs).

**Figure 3:**
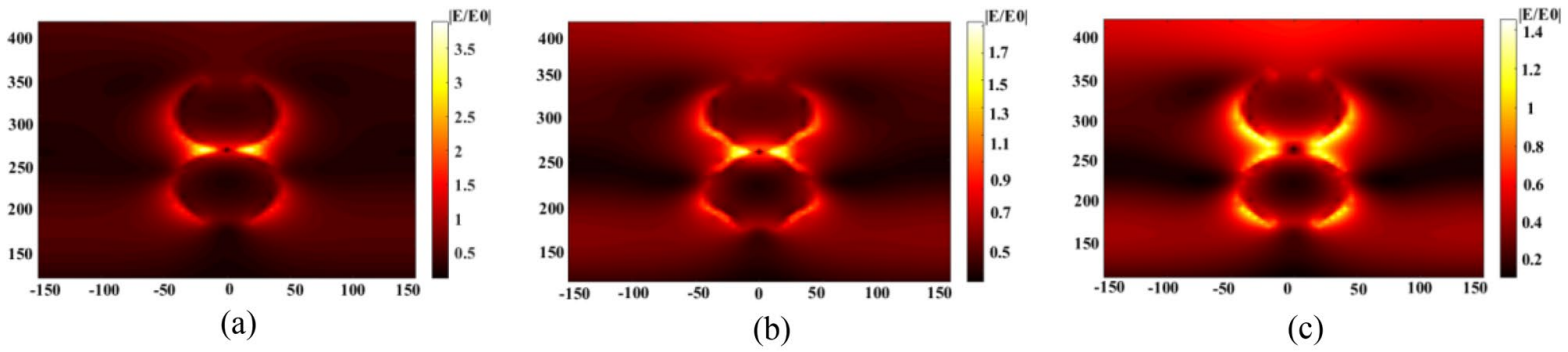
Electric field intensity profile of the CIGS/Perovskite solar cell for the optimal spherical nanostructure for different distances (**a**) 5 nm, (**b**) 10 nm, and (**c**) 15 nm).

After the analysis of Fig. 2a, it is evident that J_sc_ of the tandem structure is directly influenced by the size and geometry of the single nanoparticles (SNP) and cluster nanostructures (CNS). Our simulation results indicate that embedding nanoparticles in a cluster structure must not exceed 5 nm, as any distance beyond this threshold weakens the coupling and performance of the solar cell, as seen in Fig. 3. Additionally, absorption losses of the nanoparticles were examined during the simulation process. Figure 2b displays the maximum amount of absorption losses for particles of varying sizes.

Based on the data presented in Fig. 2a, it is evident that J_sc_ increases with the size of the individual nanoparticles. However, it is crucial to factor in the absorption losses that occur in larger nanoparticles, as shown in Fig. 2b. Therefore, it is imperative to identify the nanoparticle structure that offers minimal losses. The research suggests that the spherical, cubical, and cylindrical nanoparticles, measuring 80 nm, 60 nm, and 80 nm, respectively, are the most suitable options.

Consider the size and shape of cluster nanostructures carefully as they have a significant impact on short-circuit current density. Our observations indicate that J_sc_ increases with the size of the structure up to a certain threshold, after which it decreases. This trend is most noticeable in cubical and cylindrical nanostructures, whereas spherical nanostructures may demonstrated that the most favorable outcomes are obtained with spherical, cubical, and cylindrical nanostructures measuring 80 nm, 40 nm, and 60 nm, respectively.

To summarize, the findings indicate that while particle coupling can improve J_sc_ compared to a single nanoparticle with spherical and cylindrical geometry, using a cubical structure instead of a single nanostructure may lead to a drop in this parameter. Among the selected structures, a single cylindrical nanoparticle measuring 80 nm and a spherical nanostructure measuring 80 nm appear to achieve the highest short-circuit current densities.

As depicted in Fig. 3, it is possible to separate nanoparticles and prevent their coupling by increasing the distance between them. This eliminates the overlap of their electric fields, resulting in a decrease in field intensity. As a result, the absorption of light, current density, and overall system efficiency decrease.

**Figure 4:**
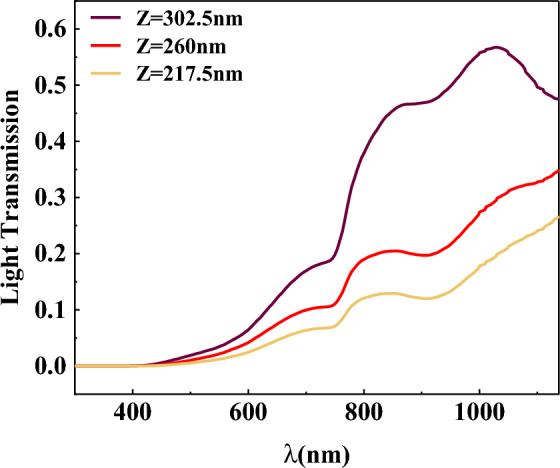
Light transmission curve if the monitor is placed in the center of CIGS layer and the centers of metal nanoparticles in the optimal spherical cluster nanostructure.

When observing Fig. 2b, may raise the question of why the absorption loss of cluster nanostructures is similar to that of a single nanoparticle, despite being calculated from the total absorption loss of two nanoparticles in CNS. However, examining the light transmission curve in Fig. 4 provides valuable insights. The transmission monitors were placed at three different locations: the center of CIGS (260 nm) and the center of optimal spherical nanostructures (217.5 nm and 302.5 nm) for this analysis.

In Fig. 4, it is evident that the light intensity inside the active layer varies. It is higher in the upper parts and gradually decreases downwards. As a result, the nanoparticles located in the lower parts of the active layer experience fewer absorption losses, while the possibility of absorption and loss inside metal nanoparticles increases with the light intensity. By placing the cluster nanostructure vertically, the bottom and top nanoparticles experience slightly lower and higher absorption losses, respectively, while the single nanoparticle located in the center of CIGS experiences slightly higher absorption losses. This leads to the total absorption losses of the cluster nanostructure being slightly higher than that of a single nanoparticle.

**Figure 5:**
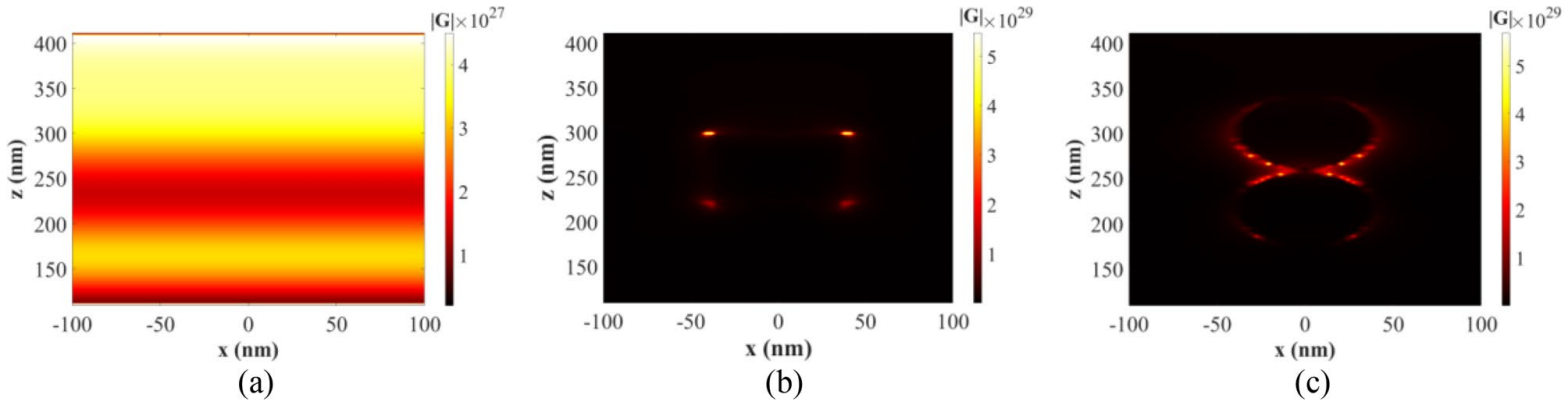
Generation rate for CIGS layer in CIGS/Perovskite tandem structure in (**a**) bare structure, (**b**) using an optimal single cylindrical nanoparticle, and (**c**) an optimal spherical nanostructure.

**Figure 6:**
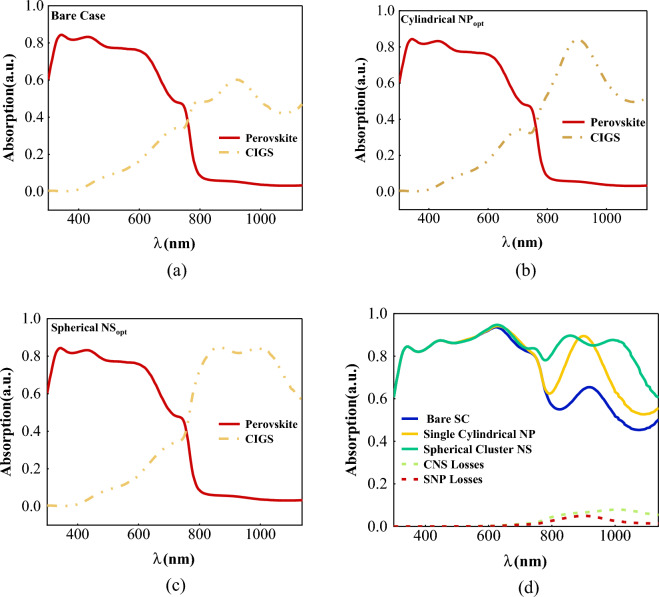
Absorption spectrum for top and bottom active layer, (**a**) at bare case, (**b**) with single cylindrical NP, (**c**) with cluster spherical NS and (**d**) total absorption of a, b, and c, as well as, absorption losses of nanoparticles.

**Figure 7:**
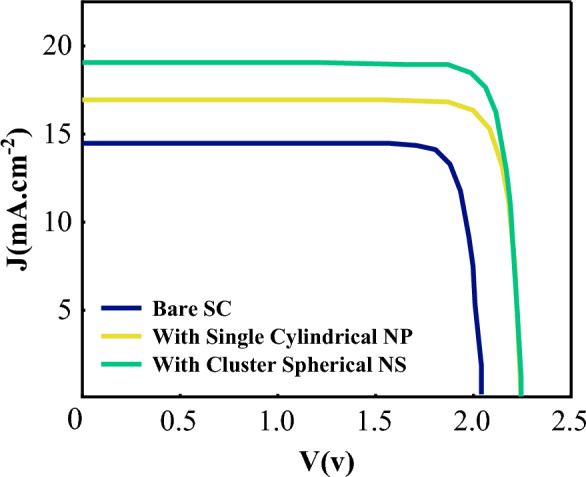
J–V characteristics curve for optimal CIGS/Perovskite solar cell, at bare case, with single cylindrical NP, and with cluster spherical NS.

The generation rate profile demonstrates that the intensity of this parameter increases in the corners of SNPs (see Fig. 5). In the case of the CNS, an improvement is observed between the top and bottom nanoparticles, leading to a significant enhancement of J_sc_ and PCE of the solar cell. A comparison was made on the tandem absorption spectrum of double-junction CIGS/Perovskite for different states after analyzing the generation rate and absorption spectrum (Fig. 6). The results revealed that perovskite solar cells exhibit weak absorption within the wavelength range of 650–800 nm. However, combining CIGS with perovskite in the tandem structure resolves this issue. Moreover, the incorporation of cluster nanostructure inside CIGS leads to an improvement in the absorption spectrum within wider spectra. As a result, the efficiency of the double-junction plasmonic CIGS/Perovskite solar cell is significantly enhanced. Figure 7 shows J–V characteristic curves. The improvement of the current density can be seen in this diagram relative to the bare structure. According to the obtained results, the current density has improved by about 23% compared to the initial structure.

Table 2 illustrates the comparison between different tandem solar cells and the improvement of PCE are shown.

**Table 2:** PCE in optimal conditions for different CIGS/Perovskite tandem solar cells.

Solar cells	PCE (%)
CIGS/perovskite SC^32^	23.1
CIGS/perovskite SC^33^	24.2
CIGS SC^22^	19.0
Perovskite SC^34^	15.4
Baseline CIGS/perovskite SC (this work)	26.35
CIGS/perovskite SC with optimal SNP (this work)	31.76
CIGS/perovskite SC with optimal CNS (this work)	35.81

## Conclusion

This research demonstrates the significant impact of coupling plasmonic nanoparticles on the performance of double-junction solar cells made of CIGS and perovskite. In order to achieve better results, cluster nanostructures with various geometries and sizes are incorporated into the center of CIGS. Using absorption spectrum, short-circuit current density, and power conversion efficiency, the optimal size and geometry for the cluster nanostructure are determined while taking into account metal nanoparticle absorption losses. To fully understand the benefits of the cluster structure and the coupling effect, it is compared with a single nanoparticle placed in the same location with the same parameters as before. The results indicate that the proposed cluster nanostructure significantly improves the absorption spectrum and power conversion efficiency of the double-junction solar cell (with a PCE_CNS_ of 35.81%) compared to the single nanoparticle (with a PCE_SNP_ of 31.76%) and the bare case (with a PCE_baseline_ of 26.35%).

It is clear that the suggested structure has immense potential to enhance the performance of multi-junction thin-film solar cells. Not only does it improve the absorption spectrum of the top active layer, but it also greatly enhances photon absorption in the bottom solar cell.

## Data Availability

The data that support the findings of this study are available from the corresponding author upon reasonable request.
